# Comparing Medium-Term Clinical Outcomes following XEN® 45 and XEN® 63 Device Implantation

**DOI:** 10.1155/2020/4796548

**Published:** 2020-03-23

**Authors:** Aitor Fernández-García, Ying Zhou, Mercedes García-Alonso, Henry D. Andrango, Francisco Poyales, Nuria Garzón

**Affiliations:** ^1^Miranza IOA Madrid, C/Galileo 104, Madrid 28003, Spain; ^2^Optometry and Vision Department, Faculty of Optics and Optometry, Complutense University of Madrid, Madrid, Spain

## Abstract

**Purpose:**

To evaluate medium-term clinical outcomes with XEN® 45 or XEN® 63 Gel Stent (Allergan, Dublin, Ireland) for treatment of primary open angle glaucoma (POAG). *Materials and Methods*. Retrospective, descriptive, and observational study involving 40 patients implanted with a XEN® 45 Gel Stent and 34 implanted with a XEN® 63 Gel Stent who had undergone POAG surgery and had been followed up and controlled between 12 and 36 months.

**Results:**

IOP dropped from 18.02 ± 5.23 mmHg preop to 13.81 ± 1.88, 14.80 ± 2.23, and 14.62 ± 1.90 at 1, 2, and 3 years after surgery (*p* < 0.001) consecutively with XEN® 45 and from 19.00 ± 6.11 mmHg preop to 15.47 ± 2.45, 14.66 ± 2.45, and 15.46 ± 2.48 at 1, 2, and 3 years after surgery (*p* < 0.001) with XEN® 63. The number of drugs used by patients to treat their glaucoma decreased after undergoing surgery in both groups. Within the XEN® 45 group, mean changes at 1 year, 2 years, and 3 years amounted to 70%, 74.3%, and 37.5%, respectively, whereas within the XEN® 63 group, the mean reduction was 75%, 79.8%, and 71.9%. When comparing the outcomes for two groups, the differences did not prove to be statistically significant. More than 90% of the procedures included in the study (using either gel-stent device) were completed without any noteworthy complications.

**Conclusion:**

POAG surgical procedures with either XEN® 45 or XEN® 63 Gel Stent implantation could be a safe and effective treatment approach.

## 1. Introduction

Glaucoma is the leading cause of global irreversible blindness [1]. This disease is a progressive optic neuropathy that leads to the death of the optic nerve's ganglion cells and the corresponding loss of visual field. Untreated or inadequately controlled glaucoma commonly results in severe vision loss, which has a tremendous socioeconomic impact [2].

Glaucoma surgery has significantly evolved in recent years with the advent of new MIGS (minimally-invasive glaucoma surgery) devices. These devices have been introduced so as to have another alternative to decrease elevated IOP and to minimize the use of topical medication, while being a technically less invasive approach than traditional glaucoma filtration surgery (trabeculectomy) [3–5].

Regarding the above mentioned MIGS, this simple surgical technique may help to prevent the effects associated with the chronic use of topical medication and to tackle the issue of low adherence to glaucoma medical treatments. The main feature of these minimally-invasive devices is that they hardly do any surgical damage [3, 6–8].

The purpose of this study is to show the outcomes obtained in patients implanted with either a XEN® 45 or a XEN® 63 Gel Stent (Allergan, Dublin, Ireland); both are MIGS devices that create a subconjunctival drainage pathway. Patient follow-up ranged between 1 and 3 years.

## 2. Materials and Methods

This is a retrospective, descriptive, and observational study involving patients with primary open-angle glaucoma (POAG) who underwent surgery with XEN gel-stent implantation, either a XEN® 45 or a XEN® 63, and who have been followed up and controlled for at least one year.

All patients belong to the Miranza IOA database and were operated by the same experienced surgeon (AF-G) between 2014 and 2017.

The inclusion criteria were as follows: patients with open-angle glaucoma who had either a XEN® 45 or a XEN® 63 Gel Stent implanted in a standalone surgery or in the context of a combined glaucoma-cataract surgery and whose medical history included at least one year of follow-up data.

The exclusion criteria were as follows: any relevant ophthalmic conditions other than glaucoma, eye trauma, or a history of eye surgery other than cataract.

Surgeries were performed in some cases due to a poor IOP control or progression despite good IOP control with several medications.

The decision to implant one stent model or the other depended on their commercial availability: The XEN® 63 was the first model marketed by the manufacturing company, and it was the one that our patients had implanted while it was commercially available. The laboratory then replaced it with the XEN® 45 and, therefore, this model was the one that was implanted thereafter.

The following metrics to assess surgical outcomes were chosen and pre- vs. postsurgical values were compared: intraocular pressure (IOP), visual-field mean defect (MD), retinal nerve fiber layer (RNFL) thickness, number of glaucoma drugs the patient was treated with, and intrasurgical or postoperative complications.

Intraocular pressure was measured by means of Goldmann tonometry, whereas visual-field mean defect was evaluated using an *Octopus* perimeter (Haag-Streit Inc., Köniz, Switzerland), G1x strategy. Regarding nerve fiber layer thickness, it was measured with the Cirrus HD-OCT platform (Carl Zeiss Meditec, Inc., Dublin, CA, USA), which relies on optical coherence tomography.

The study was approved by the Ethics Committee of the Clínico San Carlos Hospital (Madrid), and they granted us a waiver of informed consent since the study entailed no intervention except for data collection. The study is in compliance with the tenets of the Declaration of Helsinki.

### 2.1. XEN® 45 and XEN® 63

The XEN® Gel Stent Implant is a device consisting of a small hydrophilic tube that is 6 mm long and has an inner diameter of either 45 microns for the XEN® 45 or 63 microns for the XEN® 63 models and an external diameter of either 150 or 240 microns, respectively. The XEN® 63 is an older prototype with a bimanual injector that is no longer in the market.

The XEN® 45 and XEN® 63 provide 6–8 mmHg [9] and 2–3 mmHg [10] of outflow resistance at physiologic aqueous humour production rates, as predicted by the Hagen–Poiseuille equation. Both devices were made of biocompatible porcine gelatin that has been cross-linked with glutaraldehyde, so as to provide the final product with a certain rigidity [10, 11].

Subconjunctival flow creates a nonphysiological pathway for aqueous humor drainage; it is the basis of traditional trabeculectomy and aqueous shunt glaucoma surgeries. Hypotony is avoided thanks to the outflow resistance mentioned above. These Gel Stent models are typically inserted via an *ab interno* approach using a preloaded injector. This *ab interno* approach generally makes use of a clear cornea incision located opposite the implantation quadrant. For both XEN® 45 and XEN® 63 Gel Stent models, the surgery is performed under topical anesthesia, either as a standalone procedure or combined with cataract surgery.

### 2.2. Surgical Technique

The surgical technique is the same described by Fernández-García [12] for both XEN® 45 and XEN® 63 Gel Stents. They were performed under topical anesthesia, and 0.1 ml of mitomycin C as an adjunctive agent (having a concentration of 0.02 mg/ml) was introduced 5 mm away from the limbus in the area where the XEN Gel Stent was to be implanted.

According to our protocol, postoperatively, patients were treated with a combination of antibiotic, corticosteroid, and anti-inflammatory drops (moxifloxacin, dexamethasone, and bromfenac) during 5 weeks.

### 2.3. Statistical Analysis

Data analysis has been performed with the statistical software package SPSS Statistics v. 22.0 (IBM, Armonk, NY, USA).

A descriptive analysis has been carried out involving all the sociodemographic and clinical variables collected at the beginning (preoperative, baseline data), as well as the intrasurgical and postoperative data, such as postoperative complications or the need for reintervention (secondary surgeries). As for categorical variables, absolute and relative frequencies were determined; while for continuous variables, mean, standard deviation, median, and minimum and maximum values were chosen as descriptors (including the total number of valid values).

The paired *t*-test for parametric tests and the Wilcoxon and Friedman tests for nonparametric tests were used to make comparisons between subjects belonging to the same group (i.e., implanted with the same XEN Gel Stent model). On the other hand, to compare the outcomes of the two Gel Stent models, we relied on Student's *t*-test for independent samples and the Mann–Whitney *U* test for parametric and nonparametric samples, respectively. For all the quantitative variables included in this analysis, the normality assumption was tested with the Shapiro–Wilk test since each group comprised less than 50 eyes.

For all tests, the threshold for statistical significance was assumed to be *p*=0.05.

## 3. Results

### 3.1. Preoperative Data

A total of 74 eyes (39 right eyes and 35 left eyes) from 74 patients were included in this study.

Forty of them were implanted with a XEN® 45 Gel Stent and 34 of them with a XEN® 63 Gel Stent. As for the gender breakdown, 63% of all participants were female and 37% were male, while mean age was 77.31 ± 6.33 years (range: 65 to 89 years) for the XEN® 45 group and 73.36 ± 8.83 years for the XEN® 63 group (range: 55 to 85 years) (Table 1).

Regarding procedure type, 80.0% of the patients in the XEN® 45 group underwent combined surgery, while this value increased to 94.1% among the XEN® 63 patients.

### 3.2. Preoperative vs. Postoperative Result Comparison

Table 2 shows, for each follow-up visit, the pre- vs. postoperative changes in intraocular pressure (IOP), perimetry's mean deviation (MD), retinal nerve fiber layer (RNFL) thickness, and number of IOP-lowering medications required by the patients both for the XEN® 45 and the XEN® 63 patient groups, as well as the *p* value corresponding to the comparison between the two groups.

**Figure 1:**
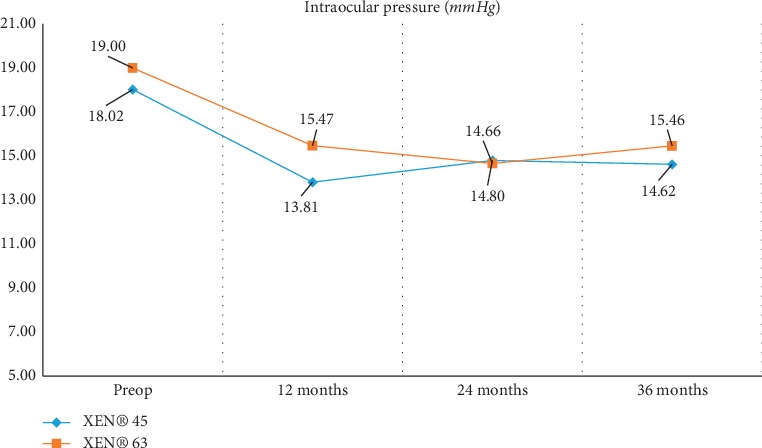
Change in mean IOPs over 12, 24, and 36 months follow-up visits for XEN® 45 and XEN® 63.

Regarding intraocular pressure, both with XEN® 45 and with XEN® 63, all decreases in IOP that occurred after device implantation turned out to be statistically significant. When comparing the two gel stent models and the IOP changes that were measured, statistically significant differences were only observed at the 12-month follow-up evaluation (*p*=0.002) (Figure 1).

In terms of perimetry's mean deviation (MD), no significant intragroup or intergroup changes were found during the years in which follow-up was performed.

As for the nerve fiber layer, a slight variation in thickness was found although it was not significant in any of the follow-up visits (which took place one, two, and three years after the procedure), neither in terms of pre- vs. postoperative changes within each group nor when comparing the two groups with each other.

**Figure 2:**
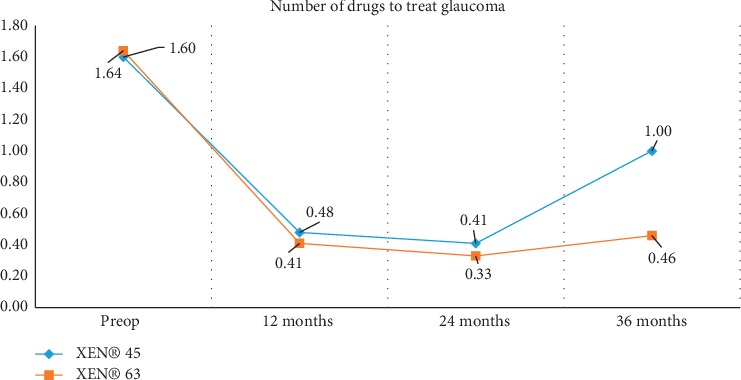
Change in number of IOP-lowering medications required by the patient over 12, 24, and 36 months follow-up visits XEN® 45 and XEN® 63.

Finally, regarding the number of drugs used by patients to treat their glaucoma, the value decreased after undergoing surgery in both patient groups. The drop remained statistically significant over all the years of follow-up. When comparing the outcomes for two groups (XEN® 45-implanted vs. XEN® 63-implanted patients), the differences did not prove to be statistically significant (Figure 2).

### 3.3. Complications

Table 3 shows the complications reported in this study.

In two of those cases, a bleb needling was required (both of them with XEN® 45 Gel Stent), and it was reconverted by means of the dry-lake technique [13].

## 4. Discussion

MIGS-type glaucoma surgery is increasingly widespread among glaucoma specialists due to the benefits it offers over other more traditional approaches, such as trabeculectomy, although not all patient cases are eligible for this less invasive technique [14]. Our study shows the evolution of a group of patients with primary open-angle glaucoma who underwent the surgical implantation of either a XEN® 45 or a XEN® 63 Gel Stent.

When evaluating the changes occurred thanks to these two implants, it is important to highlight the significant drop in intraocular pressure (mean value exceeding 18%) and the fact that this reduction was maintained throughout the 3 years of postoperative follow-up. It should be noted that IOP reduction was similar for both Gel Stent models across all follow-up visits, the only exception being the one-year evaluation, when a greater mean variation was observed in the XEN® 45 group, the difference being statistically significant (*p*=0.002).

Mansouri et al. [15] also found a significant decrease of IOP although his study focused only on the first year of postimplantation follow-up, as did De Gregorio's et al. [16], who reported in their study a 44% IOP drop. Moreover, it is worth pointing out that these two studies only assessed the XEN® 45 Gel Stent and that Mansouri's included not only patients with POAG but also pseudoexfoliative glaucoma cases. Smith et al. [17], who in his study on XEN® 45 introduced the use of mitomycin, observed a postoperative (at one year) change in intraocular pressure of approximately 33%. The changes in the IOP reported by Fernández-García et al. [12] during the first 3 years after implanting XEN® 45 were between 22.2% and 19.5%. Sheybani et al., [10] in his pilot study in patients undergoing XEN® 140 or XEN® 63 implantation combined with cataract surgery (with no use of mitomycin), also reported a mean decrease in IOP of around 30% at 12 months. Similarly, Lenzhofer et al. [18], after a 4-year follow-up of XEN® 63-implanted patients, also showed a postoperative drop in IOP of about 40%. In this context, our study seems to be the only one that compares XEN® 45 and XEN® 63 outcomes with a follow-up period as lengthy as 36-months, using MMC in all cases.

Regarding how perimetry's mean deviation (MD) has changed as a result of surgery, our study, which included between 80 and 94% of combined surgeries (depending on the patient group), reveals slight changes that are neither intragroup nor intergroup significant, which is in good agreement with Lenzhofer's [18] findings for XEN® 63 and with Fernández-García [12] for XEN® 45.

Retinal nerve fiber layer (RNFL) thickness has just been reported in a study conducted by the same group with XEN® 45. It involved 93 patients with similar results [12]. Nonetheless, the change in RNFL thickness not only remained stable over time, indicating that glaucoma was well controlled following the XEN® 45 or XEN® 63 device implantation, but it did not turn out to be statistically significant. No statistically significant differences between the two groups were found either.

Our study reveals that the number of glaucoma medications required by the patient decreases after XEN® 45 or XEN® 63 implantation surgery, and most importantly that this reduction is maintained over time for both Gel Stent models. Similar findings were reported by Karimi et al. [19] although their study focused on secondary surgeries, involving the XEN® 45 Gel Stent implantation, after failed trabeculectomy. As for the reduction in the number of IOP-lowering drugs reported in our study, within the XEN® 45 group mean changes at 1 year, 2 years, and 3 years amounted to 70%, 74.3%, and 37.5%, respectively, whereas within the XEN® 63 group, the changes turned out to be more stable across the study duration, the mean reduction being 75%, 79.8%, and 71.9% at one, two, and three years, respectively. Fernández-García et al. [12] described a greater decrease in the use of medications by patients implanted with XEN® 45 (83%, 81.8%, and 46.5%). Other authors [15–17] also observed a drop in the number of IOP-lowering medications being used although their findings are limited by their short-term (one-year) postimplantation follow-up.

It is important to note that the low complication rate occurred in our study with either of these two devices; more specifically, less than 7% of patients in each group required postsurgical needling. In two of the XEN® 45-implantation procedures a dry-lake bleb reconversion [13] was performed, while none of the XEN® 63 procedures required one. If we compare our outcomes with those published in the literature, in De Gregorio's [16] study, 2.4% of the patients required needling, while in Mansouri et al. [15] as much as 36% of the open-angle glaucoma patients had to undergo needling. Such a low rate could be due in our case not only to the surgeon's experience as well as to the consistent application of subconjunctival mitomycin prior to the procedure. It could also be affected by the 4-5 weeks of intensive corticosteroid and anti-inflammatory treatment that we prescribed as part of our postsurgical protocol, which could also prevent fibroblasts proliferation and, therefore, early fibrosis.

Bleeding in the anterior chamber occurred in less than 5% of cases (the two patient groups combined).

Other complications that have been reported in the context of XEN® 45 implantation procedures were device fracture during needling [20], endophthalmitis [21], or degradation of the device [22], but we are happy to report that none of them occurred in our case series, nor was there any procedural failure (defined as presence of a secondary IOP lowering procedure or loss of light perception) [18] during the three-year follow-up period that made it necessary to replace the gel stent. Comparing our outcomes with other studies found in the literature, De Gregorio [16] (XEN® 63 implantation procedures) reported an 11.8% failure rate, whereas Lenzhofer had 10% of failed XEN® 63 implantation surgeries [18].

Although the two devices have an inner different diameter, the similarity in the results may be because the resistance is determined by the subconjunctival bleb. The subconjunctival blebs created by the two devices are very similar.

Among our study limitations are the facts that it is retrospective and that not all cases completed the 2-year and 3-year follow-up. Another limitation lies in the fact that the study was not randomized, since gel-stent model allocation depended on their availability in that particular moment in time.

## 5. Conclusions

The pre- vs. postsurgery data collected reveal that this surgical approach is significantly effective in terms of reducing both intraocular pressure and the number of glaucoma medications required by the patient. This drop is maintained at 12, 24, and 36 months post-XEN® 45 or XEN® 63 implantation. No statistically significant differences were found between the two groups, except for the mean intraocular pressure measured one year after surgery.

More than 90% of the procedures included in the study were completed without any noteworthy complications. In at least 93% of the cases, no secondary surgery was required following the implantation of the gel stent.

These findings support the hypothesis that POAG surgical procedures with either XEN® 45 or XEN® 63 Gel Stent implantation could be a safe and effective treatment approach.

## Figures and Tables

**Table 1:** Demographic data.

	XEN® 45	XEN® 63
Number eyes	40	34
Gender (female/male) (%)	57.5/42.5	56/44
Eyes (OD/OS)	22/18	22/12
Age (years)	77.31 ± 6.33 (65 to 89)	73.36 ± 8.83 (55 to 85)
Combined surgery (cataract + glaucoma) (%)	80.0	94.1

**Table 2:** Preoperative (baseline) and postoperative (for each follow-up visit) intraocular pressure (IOP), visual field mean deviation (MD), retinal nerve fiber layer (RNFL) thickness, and number of IOP-lowering medications required by the patient for XEN® 45 and XEN® 63 Gel Stent. Significance threshold was set at 0.05.

Measurement time point	N (number of eyes) XEN® 45/XEN® 63	XEN® 45 mean value (range)	XEN® 45 *p* (vs. preop values)	XEN® 63 Mean value (range)	XEN® 63 *p* (vs. preop values)	*p* (XEN® 45 vs. XEN® 63 values)
*IOP (mmHg)*						
Preop (baseline)	40/34	18,02 ± 5,23 (10 to 40)	—	19,00 ± 6,11 (11 to 36)	—	0,459
12 months	40/34	13,81 ± 1,88 (9 to 18)	≤0.001	15,47 ± 2,45 (20 to 9)	≤0.001	0,002
24 months	37/30	14,80 ± 2,23 (11 to 21)	≤0.001	14,66 ± 2,45 (7 to 20)	≤0.001	0,827
36 months	30/30	14,62 ± 1,90 (12 to 18)	≤0.001	15,46 ± 2,48 (9 to 20)	≤0.001	0,191

*Visual field-Mean deviation (MD)*						
Preop (baseline)	40/30	9,97 ± 6,12 (0,6 to 23,10)	—	10,42 ± 7,16 (-1,2 to 25,70)	—	0,777
12 months	32/30	8,98 ± 6,80 (0,0 to 24,60)	0,679	8,01 ± 5,56 (0,7 to 18,30)	0,549	0,606
24 months	30/29	10,57 ± 6,88 (1,3 to 22,80)	0,375	8,64 ± 5,94 (0,5 to 21,80)	0,067	0,353
36 months	30/24	9,10 ± 5,52 (0,3 to 16,20)	0,700	7,59 ± 6,36 (-2,0 to 18,8)	0,685	0,437

*Nerve fiber layer (microns)*						
Preop (baseline)	39/30	63,94 ± 11,42 (36 to 89)	—	59,58 ± 14,91 (36 to 89)	—	0,195
12 months	20/30	61,40 ± 10,13 (41 to 83)	0,881	62,50 ± 8,56 (44 to 79)	0,501	0,721
24 months	20/28	57,92 ± 8,15 (47 to 74)	0,064	62,15 ± 10,12 (50 to 92)	0,221	0,217
36 months	20/24	61,60 ± 10,09 (49 to 87)	0,924	64,81 ± 13,44 (41 to 94)	0,383	0,418

*Number of glaucoma medications*						
Preop (baseline)	40/34	1,60 ± 0,94 (0 to 3)	—	1,64 ± 0,77 (0 to 3)	—	0,854
12 months	40/34	0,48 ± 0,78 (0 to 2)	≤0.001	0,41 ± 0,78 (0 to 2)	≤0.001	0,696
24 months	35/30	0,41 ± 0,71 (0 to 2)	≤0.001	0,33 ± 0,71 (0 to 2)	≤0.001	0,672
36 months	36/30	1,00 ± 0,87 (0 to 2)	≤0.001	0,46 ± 0,81 (0 to 2)	≤0.001	0,059

**Table 3:** Complications reported during the study.

Complications	XEN® 45	XEN® 63
Cases without any complication (%)	92.5	94.1
Procedures required just one attempt to successfully deploy the gel stent (%)	97.5	97.05
Bleeding occurred in the anterior chamber (%)	5	2.95
Postsurgical bleb needling required (%)	5	6.9
Devices replaced during the 36 months after surgery	0	0

## Data Availability

Other researchers can access the data supporting the conclusions of the study by sending an email to the corresponding author.
